# The LspC3–41I restriction-modification system is the major determinant for genetic manipulations of *Lysinibacillus sphaericus* C3–41

**DOI:** 10.1186/s12866-017-1014-6

**Published:** 2017-05-19

**Authors:** Pan Fu, Yong Ge, Yiming Wu, Ni Zhao, Zhiming Yuan, Xiaomin Hu

**Affiliations:** 1 Wuhan Institute of Virology, Chinese Academy of Sciences, Wuhan, 430071 China; 2 University of Chinese Academy of Sciences, Beijing, 100039 China

**Keywords:** *Lysinibacillus sphaericus*, R-M systems, CFE, Methylation, LspC3–41I

## Abstract

**Background:**

*Lysinibacillus sphaericus* has been widely used in integrated mosquito control program and it is one of the minority bacterial species unable to metabolize carbohydrates. In consideration of the high genetic conservation at genomic level and difficulty of genetic horizontal transfer, it is hypothesized that effective restriction-modification (R-M) systems existed in mosquitocidal *L. sphaericus*.

**Results:**

In this study, six type II R-M systems including LspC3–41I were predicted in *L. sphaericus* C3–41 genome. It was found that the cell free extracts (CFE) from this strain shown similar restriction and methylation activity on exogenous *Bacillus*/*Escherichia coli* shuttle vector pBU4 as the *H*aeIII, which is an isoschizomer of BspRI. The Bsph_0498 (encoding the predicted LspC3–41IR) knockout mutant Δ0498 and the complement strain RC0498 were constructed. It was found that the unmethylated pBU4 can be digested by the CFE of C3–41 and RC0498, but not by that of Δ0498. Furthermore, the exogenous plasmid pBU4 can be transformed at very high efficacy into Δ0498, low efficacy into RC0498, but no transformation into C3–41, indicating that LspC3–41I might be a major determinant for the genetic restriction barrier of strain C3–41*.* Besides, *lspC3–41IR* and *lspC3–41IM* genes are detected in other two strains besides C3–41 of the tested 16 *L. sphaericus* strains, which all belonging to serotype H5 and MLST sequence type (ST) 1. Furthermore, the three strains are not horizontal transferred, and this restriction could be overcome by in vitro methylation either by the host CFE or by commercial methytransferase M. *Hae*III. The results provide an insight to further study the genetic restriction, modification and evolution of mosquitocidal *L. sphaericus,* also a theoretical basis and a method for the genetic manipulations of *L. sphaericus.*

**Conclusions:**

LspC3–41I is identified as the major determinant for the restriction barrier of *L. sphaericus* C3–41*.* Only three strains of the tested 16 *L. sphaericus* strains, which all belonging to serotype H5 and ST1 by MLST scheme, contain LspC3–41I system. Two different methods can be used to overcome the restriction barrier of the three isolates to get transformants efficiently: 1) to methylate plasmid DNA prior to the electroporation; and 2) to delete the major restriction endonuclease encoding gene *lspC3–41IR*.

## Background

*Lysinibacillus sphaericus* is a spore-forming bacterium widely distributed in soil and other environment. *L. sphaericus* can metabolize a large variety of organic compounds and amino acids but cannot utilize polysaccharide because of absence of key enzymes in glycolysis [1]. The classifications of *L. sphaericus* were mainly based on flagellar agglutination and DNA typing, 49 serotypes [2] and five major DNA groups (I to V) [3] have been identified. Among them, nine serotypes (H1, H2, H3, H5, H6, H9, H25, H26, and H48) can produce different types of insecticidal proteins against mosquito larvae: the binary toxin (Bin) and two-component toxin (Cry48 and Cry49) formed during sporulation, and mosquitocidal toxins (Mtx toxins) produced during the vegetative growth [4–7]. Several typing methods, e.g. 16S rRNA and MLST analysis, revealed that the mosquitocodal *L. sphaericus* are highly conserved. [8, 9]. In addition, comparative genomics revealed that toxic strains form a well-defined lineage that could be considered as a separate species from non-toxic strain [10].

Even *L. sphaericus* has been successfully applied as bacterial pesticide for mosquito larvae control, the relative narrow spectrum and the occurrence of high-level resistance in target mosquitoes in field hindered its further utilization in integrated mosquito management program [11–13]. Therefore, there is a great demand to construct genetic engineered *L. sphaericus* with broader active spectrum and higher activity. Besides, being an archaic organism and one of the minor bacterial species unable to metabolize carbohydrates, *L. sphaericus* is regarded as a good model strain for studying bacterial genetic evolution and metabolism regulation. However, the difficulty of horizontal transfer of foreign DNA and the unavailability of genetic manipulations in many high toxic *L. sphaericus* strains limited the related study [14]. Moreover, genomic analysis among some sequenced strains revealed a high DNA homology, especially the identical binary toxin genes identified from the strains isolated from different continents, and the reason for this high conservation remains unclear. We make a hypothesis that after one *L. sphaericus* lineage acquired mosquitocidal toxin genes, it developed restriction-modification (R-M) systems which prevent the invasion of foreign DNA.

To maintain species identity, most bacteria encode R-M systems to prevent the invasion of foreign DNA [15, 16]. The R-M systems usually consist of a DNA methyltransferase (MTase) and a restriction endonuclease (REase), which serve to methylate self DNA and restrictly cleave invading unmethylated DNA with the same specificity sequence, respectively [16]. The R-M system can build complex genetic barriers for unrecognized DNA, resulting in the difficulty for genetic manipulations, e.g. transformation, transfection and conjugation. There are four R-M system groups (types I to IV), classfied based on their enzyme compositions, cofactors and activity modes and of which the type II R-M system is the most abundant [17]. Most of the Type II R-M system consist of separate REase and MTase, functioned as counterpart pair by recognizing the same and normally palindromic DNA sequence [18, 19].

*L. sphaericus* C3–41 is a commercialized highly toxic strain isolated from a mosquito breeding site in China in 1987. A C3–41 liquid formulation (Jianbao®) has been successfully applied for urban mosquito larvae control for more than two decades in China and good control efficacy has been recorded. The complete genome of C3–41 has been sequenced and the molecular basis of toxin synthesis and metabolism regulation have been elucidated [20, 21]. In this study, six type II R-M systems were predicted in *L. sphaericus* C3–41, of which LspC3–41I was found to play a key role in the genetic manipulations and identified as a major determinant for the genetic restriction of *L. sphaericus*. The study helps for understanding of the biologial restriction and modification phenomenon and for developing better genetic engineering tools and techniques for *L. sphaericus*.

## Methods

### Bacterial strains, plasmids, and culture conditions

Sixteen *L. sphaericus* strains, representing 13 sequence types (STs) by MLST scheme [9], were used in this study (Table 1). *E. coli* JM109 was used for sub-cloning. The *Bacillus*/*Escherichia coli* shuttle vector pBU4 was used as the exogenous plasmid DNA for restriction and methylation analysis. pXK and pMarC333 were used as the source of kanamycin resistance gene (*kan*) and the spectinomycin resistance gene (*spc*), respectively [22, 23]. pRN5101, a temperature sensitive suicide vector, was used for recombinant for gene disruption [24]. The *L. sphaericus* and the *E. coli* strains were grown in Luria-Bertani (LB) medium at 30 °C and 37 °C, respectively. The antibiotic concentrations for bacterial selection were as follows: 100 μg/ml ampicillin and 50 μg/ml kanamycin for *E. coli*; 10 μg/ml kanamycin, 5 μg/ml erythromycin, 100 μg/ml spectinomycin, and 10 μg/ml tetracycline for *L. sphaericus*.

**Table 1:** Occurence of LspC3–41I system and the transformation capability of *L. sphaericus*

Strains	Serotype^a^	Sequence type (ST)^b^	PCR results^c^ for		Transformation efficiency (cfu μg^-1^ plasmid DNA)			
			*lspC3–41IR*	*lspC3–41 IM*	Untreated	*M. Hae*III	C3–41 CFE	Recipient CFE
C3–41	H5	1	+	+	0	2.2 × 10^5^	2.4 × 10^5^	2.4 × 10^5^
2362	H5	1	+	+	0	1.1 × 10^5^	4.6 × 10^4^	4.2 × 10^4^
1593	H5	1	+	+	0	1.9 × 10^5^	7.0 × 10^4^	5.5 × 10^4^
47-6b	H6	2	-	-	0	0	0	0
2297	H25	4	-	-	3.6 × 10^4^	ND	ND	ND
IAB872	H48	5	-	-	0	ND	0	0
IAB881	H3	6	-	-	0	ND	0	0
NRS1693	H2	7	-	-	7.4 × 10^5^	ND	ND	ND
2314-2	H9	8	-	-	0	ND	0	0
LP1-G	H3	9	-	-	0	0	0	0
Bs-197	H1	10	-	-	7	ND	8	7
2173	H26	11	-	-	0	0	0	0
Cok31	H9	12	-	-	0	ND	0	0
SSII-1	H2	13	-	-	0	ND	0	0
KellenQ	H1	14	-	-	0	ND	0	0
Dak614	H4	15	-	-	0	ND	0	0

^a^Serotype is as determined by [2]

^b^ST is as determined by [9]

^c^+, present; -, absent

ND means not determined

### Cell free extracts (CFE) preparation and restriction assay

*L. sphaericus* strains were grown in LB at 30 °C with shaking at 200 rpm until mid-exponential phase. The cells were harvested and washed twice with deionized water, and resuspended in one-third volume of TNM buffer [100 mM Tris-HCl (pH 7.5), 50 mM NaCl and 5 mM MgCl_2_]. Bacterial cell suspensions were sonicated on ice using ultrasonic processor at tapered microtip (pulse on 5 s and pulse off 5 s; totally 30 m) and then centrifuged at 12,000 rpm for 5 min at 4 °C. Supernatants as CFE were collected, and stored at −70 °C. For restriction assays, CFE was diluted 1:100 in TNM buffer and aliquots of 20 μL CFE were mixed with 500 ng plasmid and incubated for 2 h at 37 °C. The reaction mixture was analyzed by agarose gel electrophoresis and Southern blot analysis.

### In vitro methylation

In vitro methylation was performed by treated with CFE or commercial MTase. 5 μg plasmid DNA was mixed with 20 μL CFE in a reaction buffer with TNM buffer (50 μL TNM buffer was added into a 100 μL in vitro methylation reaction), 30 mM EDTA (pH 8.0) and 200 μM SAM (S-adenosylmethionine), of which the EDTA was used to inhibit the restriction of CFE, and SAM to provide methyl. The methylation with commercial MTase M. *Hae*III (New England Biolabs) was processed according to the recommended protocol by the manufacturer. As a control, plasmid DNA was mock treated in a reaction mixture without CFE or M. *Hae*III. All samples were incubated at 37 °C for 2 h. M. *Hae*III was heat inactivated at 65 °C for 20 min and removed by phenol-chloroform-isoamyl alcohol extraction as described previously [25], then analyzed by agarose gel electrophoresis.

### Creation of *L. sphaericus* mutants

All oligonucleotides used in this study are listed in Table 2. For disruption of the predicted *lspC3–41IR* gene (Bsph_0498), an upstream (0498-A) and downstream (0498-B) fragment of Bsph_0498 were amplified from the *L. sphaericus* genome using primer pairs 0498LA-F/0498LA-R and 0498RA-F/0498RA-R, respectively. The *kan* gene was amplified from plasmid pXK using primer pair Kan-F/Kan-R as described [22]. The 0498-A, *kan*, and 0498-B fragments were digested with *Sal*I/*Bam*HI, *Bam*HI/*Kpn*I, and *Kpn*I/*Nhe*I, respectively, and cloned between the *Sal*I and *Nhe*I sites in the temperature-sensitive plasmid pRN5101, yielding plasmid pRN-M0498. Following in vitro methlylation using the commercial methyltransferase M. *Hae*III, pRN-M0498 was electroporated into *L. sphaericus* C3–41 to make the mutant Δ0498. To complement Bsph_0498, a recombinant plasmid pRN-C0498 was constructed which contains the intact Bsph_0498 including its native promoter (*P*_*bspR*_) and a *spc* gene and the flanking sequences of *amyE* (Bsph_1204) as described previously [22]. Following in vitro methlylation using the commercial MTase M. *Hae*III, pRN-C0498 was electroporated into *L. sphaericus* mutant Δ0498.

**Table 2:** Oligonucleotides used in this study

Oligonucleotide	Sequence (5’ to 3’)^a^
0498LA-F (SalI)	CCCGTCGACGAAAGTGCGTCCAGCGTAT
0498LA-R (BamHI)	CCCGGATCCTTTGCTCTGCACGTTCTGC
0498RA-F (KpnI)	CCCGGTACCACTTCGCTCAAAATAATCCTAAT
0498RA-F (NheI)	CCCGCTAGCACCTAAGTCTAAACCTCCACAAC
Kan-F (BamHI)	CCCGGATCCGAACCATTTGAGGTGATAGG
Kan-R (KpnI)	CCCGGTACCGGTACTAAAACAATTCATCCAG
0498-F	GGAAGAACATCTGCTGGAA
0498-R	AGCTACGATAATTTAGTGCC
0497-F	TTTGTGGCTGAGAATGTTAA
0497-R	GTATTGGTGCTTGTCTACCTG
SpcR-F (KpnI)	CCCGGTACCAGAGTTGGTAGCTCTTGATCCG
SpecR-R (EcoRI)	CCCGAATTCTTTAAAAGTAAGCACCTGTTATTC
amyLA-F (SalI)	CCCGTCGACGAATAATGGGCAAATTCAGTGG
amyLA-R (BamHI)	CCCGGATCCAGAAGCGTCTTGTTCTTGACGA
amyRA-F (EcoRI)	CCCGAATTCATTTCACAAAGAAAATAGCA
amyRA-R (NheI)	CCCGCTAGCGGATGTGATTTACCAAAGTCT
C0498-F (BamHI)	CCCGGATCCGTATTCCAGACTGTTTGGAGCT
C0498-R (KpnI)	CCCGGTACCCTCTATCTAAATCAGGAGCAGGTT

^a^Restriction enzyme recognition sites are underlined

The *L. sphaericus* mutant Δ0498 and complement strain RC0498 were generated by homologous recombination according to heat treated method described previously based on the temperature sensitive character of pRN5101 [22]. The correct mutant and complement strains were screened by resistance and confirmed by PCR analysis.

### DNA probe labeling and hybridizations

A standard procedure described before was carried out for Southern blot analysis [25]. Briefly, the purified DNA were separated on 0.8% agarose gel, and blotted to a positively-charged nylon membrane. The plasmid pBU4 was first linearized with *Hae*III and then labeled with DIG to be used as DNA probe. The DNA labeling, hybridization, and detection were carried out using DIG High Prime DNA labeling and detection starter kit I (Roche), according to the manufacturer’s instructions.

### Measurement of plasmid transformation efficiency

The competent cells of *L. sphaericus* strains were prepared as the method described before [26]. To measure the transformation efficiency, 10 ng methylated or unmethylated shuttle vector pBU4 was added to 100 μL competent cells of *L. sphaericus* C3–41 or its derivative mutants and kept on ice for 30 min. The vector was introduced into competent cells of *L. sphaericus* strains by electroporation (2.5 kV, 25 μF and 200 Ω), and 1 ml LB was immediately added into the electroporated cells and incubated for 2 h at 30 °C, 100 rpm. The transformants were selected on LB solid plates with 10 μg/mL tetracycline and incubated overnight. The number of the colonies was counted, and the transformation efficiency was presented as transformants/μg. The data were expressed as the mean transformation efficiency from three independent experiments.

## Results and discussion

### Six R-M systems were predicted in *L. sphaericus* C3–41

**Fig. 1:**
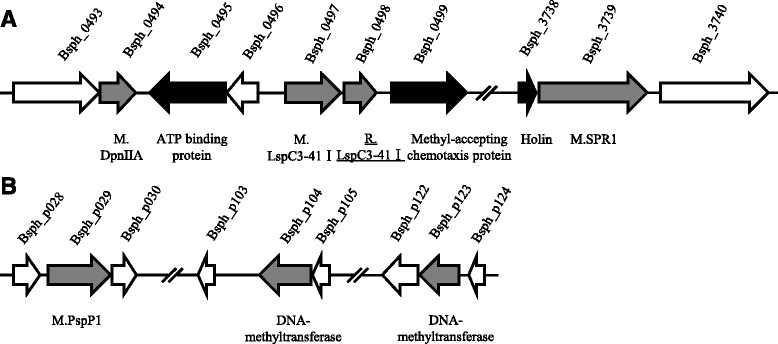
Organization of the predicted gene clusters related with restriction-modification system in the genomic region of *L. sphaericus* C3–41. Three systems (Bsph_0493-Bsph_0495, Bsph_0497-Bsph_0499 and Bsph_3738-Bsph_3740) are located on chromosome (**a**), whereas the other three (Bsph_p028-Bsph_p030, Bsph_p103-Bsph_p105 and Bsph_p122-Bsph_p124) are located on the large plasmid pBsph (**b**). The predicted endonuclese R. LspC3–41I encoding by Bsph_0498 was underlined

REBASE analyses against the whole genome sequences of *L. sphaericus* C3–41 exhibited the presence of six gene clusters related with type II R-M systems (Fig.1), in which three (Bsph_0493-Bsph_0496, Bsph_0497-Bsph_0499 and Bsph_3738-Bsph_3740) are located on chromosome, whereas the other three (Bsph_p028-Bsph_p030, Bsph_p103-Bsph_p105 and Bsph_p122-Bsph_p124) on a large plasmid pBsph. It was found that Bsph_0497, Bsph_0498 and Bsph_0499 encode proteins of a complete R-E system, of which Bsph_0497 and Bsph_0498 were predicted to encode MTase and REase, showing 61% and 33% identities to the corresponding components (M. BspRI and R. BspRI) of BspRI system, respectively. BspRI is an isoschizomer of *Hae*III, both belongs to the PD-(D/E) XK nuclease superfamily. The M. BspRI and R. BspRI pair recognize the same double-stranded sequence GGCC, of which M. BspRI causes specific methylation and protects the DNA from cleavage by its counterpart R. BspRI [27, 28]. There are MTases but no REase homologs predicted in the other five gene clusters related with type II R-M systems. When MTases exist alone, they were believed to be mainly involved in the functions related with regulation other than with restriction barrier [29]. This suggested that Bsph_0498 (named *lspC3–41IR*), encoding the putative R. LspC3–41I, might be an important determinant for the restriction barrier of *L. sphaericus* C3–41.

### pBU4 was digested by CFE of *L. sphaericus* C3–41

**Fig. 2:**
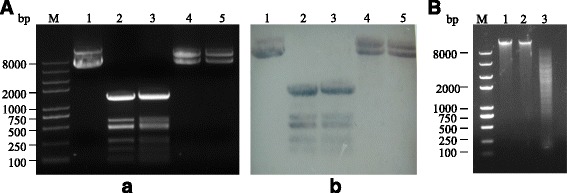
The restriction and methylation role of *L. sphaericus* C3–41 CFE. **a** Restriction profile of pBU4 (*a*) and Southern blot analysis of the restriction of pBU4 using linear pBU4 as the probe (*b*). Lane 1, pBU4 mock treated; lane 2, pBU4 digested by *Hae*III; lane 3, pBU4 digested by C3–41 CFE; lane 4, pBU4 methylated with C3–41 CFE; lane 5, pBU4 methylated with C3–41 CFE and then digested by *Hae*III. **b** Restriction assays of *L. sphaericus* C3–41 chromosome. Lane 1, chromosome mock treated; lane 2, chromosome digested by *Hae*III; lane 3, chromosome digested by *Hin*dIII. M: DNA marker

To investigate if the R-M systems of *L. sphaericus* C3–41 are functional, the *E. coli-Bacillus* shuttle vector pBU4 isolated from *E. coli* JM109 was incubated with the CFE of *L. sphaericus* C3–41. Furthermore, since R. LspC3–41I is the only predicted REase of C3–41 and it is an isoschizomer of *Hae*III, pBU4 was also incubated with commercial restriction endonuclease *Hae*III as a control. Restriction profile and Southern blot analysis showed that plasmid mixed with CFE was completely digested, displaying the similar profile with that treated with commercial restriction endonuclease *Hae*III (Fig. 2a). The result suggested that the R-M systems of C3–41, probably R. LspC3–41I, played a restriction role on the exogenous DNA. In addition, it was observed that the genomic DNA of C3–41 can be digested by *Hind*III but not by *Hae*III (Fig. 2b). This suggested that the intrinsic DNA have been methylated by the M. LspC3–41I and thus resistant to *Hae*III as they recognize the same site. The results confirmed that CFE from *L. sphaericus* could digest exogenous DNA but not intrinsic DNA due to the strain specific modification systems.

### **Δ**0498 cannot digest unmethylated pBU4

**Fig. 3:**
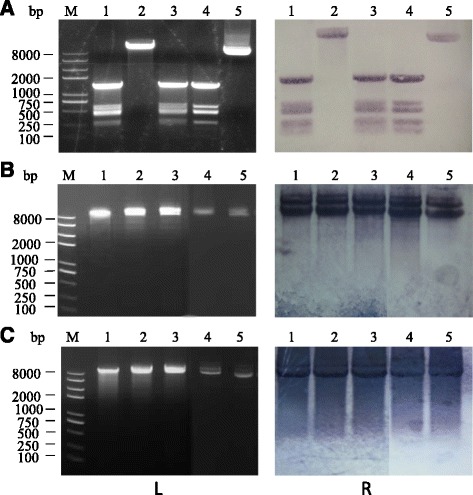
The effect of Bsph_0498 (encoding LspC3–41I) on the restriction role of *L. sphaericus* C3–41 CFE. Untreated and pre-treated plasmid pBU4 was incubated with CFE and then subjected to restriction assays, and the reaction mixture was analyzed by agarose gel electrophoresis as show in the left three pictures (L) and Southern blot analysis as show in the right three pictures (R). **a** untreated pBU4. Lane 1, C3–41; lane 2, Δ0498; lane 3, RC0498; lane 4, *Hae*III digested; lane 5, Untreated. **b** pBU4 methylated with C3–41 CFE. Lane 1, C3–41; lane 2, Δ0498; lane 3, RC0498; lane 4, *Hae*III digested; lane 5, Untreated. **c** pBU4 methylated with MTase M. *Hae*III. Lane 1, C3–41; lane 2, Δ0498; lane 3, RC0498; lane 4, *Hae*III digested; lane 5, Untreated. M: DNA marker

A mutant with Bsph_0498 knockout (Δ0498) and its complement strain RC0498 were constructed. Restriction profile and Southern blot showed that the unmethylated pBU4 can be digested by C3–41 CFE but not by Δ0498 CFE, whereas the CFE of the complement strain RC0498 restored the cleaving capability against pBU4 and showed the same digested profile with the wild (Fig. 3a). Nevertheless, after methylated by CFE or M. *Hae*III, the plasmid became resistant to the CFE of all the three strains (Fig. 3b and c). The results suggested that R. LspC3–41I should be a major determinant for the restriction barrier against exogenous DNA in *L. sphaericus* C3–41.

### Both methylated and unmethylated pBU4 can be transformed efficiently into Δ0498

**Fig. 4:**
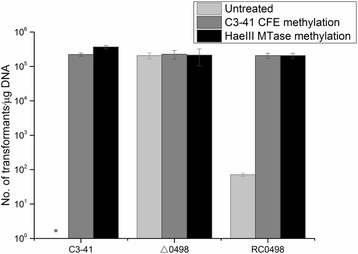
The transformation frequencies of pBU4 into *L. sphaericus* C3–41 and its derivate mutants. Light gray column: pBU4 untreated; deep gray column: pBU4 methylated with C3–41 CFE; black column: pBU4 methylated with MTase M. *Hae*III. *, no transformant observed

The methylated and unmethylated pBU4 were electroporated into C3–41, Δ0498 and RC0498. As expected, the unmethylated pBU4 was successfully transferred into △0498 but not into C3–41, with similar transformation efficiency to that when the methylated pBU4 transferred into C3–41 (ca. 2 × 10^5^ transformants/μg DNA) (Fig. 4). The unmethylated plasmid was also able to be transformed into the complement strain RC0498, but in a much lower efficiency (ca. 7 × 10 transformants/μg DNA). Therefore, the complementation of the R. LspC3–41I cannot completely build a barrier for the foreign DNA. The reason remains unclear, probably due to the low copies and instability of the pRN-vector. All the three strains, C3–41, Δ0498 and RC0498 can accept methylated pBU4 with C3–41 CFE or M. *Hae*III, with similar efficiencies (ca. 2 × 10^5^ transformants/μg DNA) (Fig. 4). The results suggest that R. LspC3–41I is indeed the major determinant for the restriction barrier of C3–41 and in vitro methylation can overcome the restriction barrier and increase the transformation efficiency of *L. sphaericus* C3–41 and its derived mutants.

### LspC3–41I system is only found in *L. sphaericus* belonging to the serotype H5 and MLST type ST1

**Fig. 5:**
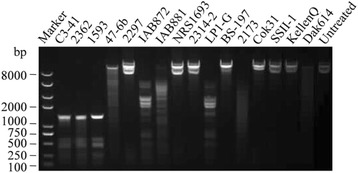
The restriction profile of pBU4 by the CFE of 16 *L. sphaericus*. Plasmid DNA was mixed with *L. sphaericus* CFE and the reaction mixture was analyzed by agarose gel electrophoresis. M: DNA marker

The unmethylated pBU4 was mixed with the CFE of 16 *L. sphaericus* strains, representing 10 serotypes and 13 MLST sequence types [9], respectively. The agarose gel displayed that the CFE of nine strains can partially or completely digest pBU4, whereas the other seven cannot (Fig. 5). Besides C3–41, only 2362 and 1593, all belonging to the serotype H5 and MLST ST1, are PCR positive to *lspC3–41IR* and *lspC3–41IM* genes. Furthermore, using the host CFE or M. *Hae*III to treat pBU4 can make 2362 and 1593 become transformable as well as C3–41 (Table 1). Based on the reported complete genome sequence of 2362 (Genbank accession No. NZ_CP015224 [30], three type II restriction-modification systems were predicted in REBASE. It was found that A2J09_14565 and A2J09_14570 encode proteins of a complete R-E system, showing 34% and 61% identities to R. BspRI and M. BspRI of BspRI system, respectively. It is therefore deduced that like in C3–41, BspRI may be the major determinant for genetic manipulations of 2362. Although several other genetic manipulations of 1593 and 2362 have been reported, e.g. protoplast transformation method [31] and conjugational transfer from *E. coli* using some special vectors [32]. However, these methods are unstable, time-consuming and low efficient, whereas in vitro methylation by CFE or M. *Hae*III is easy handled and can make the foreign DNA to be more acceptable to the host with high efficiency. pBU4 could also be digested by the CFE of 47-6b, IAB872, IAB881, LP1-G, 2173 and Dak614, however, the incubation with their CFE cannot overcome the transformation difficulty. It is possible that some other complicated genetic barrier is presented in these strains or some unknown cofactors are necessary for the action of methylation function. Although four strains unable to digest pBU4 in vitro by self CFE (2314–2, Cok31, SSII-1, KellenQ), they have no transformation capability. One possibility is that there are other unknown R-M systems in these strains which cannot recognize the sequence of pBU4 in the tested buffer. Indeed, the presence of different types of restriction endonucleases in *L. sphaericus* was reported. For instance, *Bae*I recognizes (10/15) ACNNNNGTAYC (12/7) requiring both Mg^2+^ and AdoMet to cleave DNA [33], *Bsi*I is not an isoschizomer of any known restriction endonucleases which recognizes non-palindromic nucleotide sequence C1TCGTG [34]. Besides R-M systems, a recent report clustered regularly interspaced short palindromic repeat (CRISPR)-Cas can also prevent the invasion of foreign DNA by acquired immunity [35, 36]. Although most *L. sphaericus* are hard to be genetically manipulated, there are three strains (2297, NRS1693 and Bs-197) which are naturally deficient in restriction enzymes have the transformation capability without foreign DNA modification (Table 1). This is consistent with our previous experiences that 2297 and NRS1693 are capable of genetic manipulations, not only for pBU4 but also for many other heterogenous recombinant plasmids.

## Conclusion

In this study, six type II R-M systems were predicted in *L. sphaericus* C3–41 genome, of which *lspC3–41IR* (Bsph_0498) is the only predicted REase encoding gene. The CFE from *L. sphaericus* C3–41, on one hand can digest exogenous *Bacillus*/*E. coli* shuttle vector pBU4, on the other hand can methylate the same plasmid in vitro in a methyl-buffer. LspC3–41I was identified as the major determinant for the restriction barrier of *L. sphaericus* C3–41*.* Only three strains of the tested 16 *L. sphaericus* strains, which all belonging to serotype H5 and ST1 by MLST scheme, contain LspC3–41I system. Two different methods can be used to overcome the restriction barrier of the three isolates to get transformants efficiently: 1) to methylate plasmid DNA prior to the electroporation; and 2) to delete the major restriction endonuclease encoding gene *lspC3–41IR*. The function of the other five R-E systems in C3–41 remains unclear. Our previous studies have showed that the genomes of mosquitocidal *L. sphaericus* isolates are highly conserved, and hypothesized that the timescale of divergence between mosquitocidal and non-mosquitocidal strains of *L. sphaericus* should be large [9]. We have also indicated that the mosquitocidal toxin genes were acquired by horizontal gene transfer (HGT) due to the relationship between the mobile genetic elements (MGEs) and the toxin genes [21]. It is hypothesized that the R-M systems in *L. sphaericus* be developed after one lineage acquired mosquitocidal genes by HGT, and then genetic barriers be build for the toxic *L. sphaericus* to accept foreign DNA and therefore make them conserved.

## Abbreviations

CFE: Cell free extracts
HGT: Horizontal gene transfer
*L. sphaericus*: *Lysinibacillus sphaericus*
MGEs: Mobile genetic elements
MLST: Multi-locus sequencing typing
R-M: Restriction-modification
